# Adiabatic spin-lock preparations enable robust in vivo cardiac *T*_1*ρ*_-mapping at 3T

**DOI:** 10.1109/EMBC48229.2022.9871870

**Published:** 2022-07

**Authors:** Chiara Coletti, Joao Tourais, Telly Ploem, Christal van de Steeg-Henzen, Mehmet Akçakaya, Sebastian Weingärtner

**Affiliations:** 1 Department of imaging physics, Delft University of Technology (TU Delft), Delft, The Netherlands.; 2 HollandPTC consortium – Erasmus Medical Center, Rotterdam, Holland Proton Therapy Centre, Delft, Leiden University Medical Center (LUMC), Leiden and Delft University of Technology, Delft, The Netherlands; 3 Department of electrical and computer engineering and Center for Magnetic Resonance Research, University of Minnesota, Minneapolis, United States of America.

## Abstract

Magnetic Resonance Imaging (MRI) is the clinical gold standard for the assessment of myocardial viability but requires injection of exogenous gadolinium-based contrast agents. Recently, *T*_1*ρ*_-mapping has been proposed as a fully non-invasive alternative for imaging myocardial fibrosis without the need for contrast agent injection. However, its applicability at high fields (> 1.5 T) is hindered by susceptibility to MRI system imperfections, such as inhomogeneities in the $B_{0}$ and $B_{1}^{+}$ fields.

In this work we propose a single breath-hold ECG-triggered single-shot bSSFP sequence to enable *T*_1*ρ*_-mapping in vivo at 3T. Adiabatic *T*_1*ρ*_ preparations are evaluated to reduce $B_{0}$ and $B_{1}^{+}$ sensitivity in comparison with conventional spin-lock (SL) modules. Numerical Bloch simulations were performed to identify optimal parameters for the adiabatic pulses. Experiments yield *T*_1*ρ*_ values in the myocardium equal to 148.13 ± 54.08 ms for the best adiabatic preparation and 16.01 ± 20.75 ms for the reference non-adiabatic SL, with 26.91% against 89.74% relative difference in *T*_1*ρ*_ values across two shimming conditions. Both phantom and in vivo measurements show increased myocardium/blood contrast and improved resilience against system imperfections compared to non-adiabatic *T*_1*ρ*_ preparations, enabling the use at 3T.

**Clinical relevance:**

Adiabatically-prepared *T*_1*ρ*_-mapping sequences form a promising candidate for non-contrast evaluation of ischemic and non-ischemic cardiomyopathies at 3T.

## I. INTRODUCTION

Cardiac Magnetic Resonance Imaging (MRI) is the main clinical tool for tissue characterization of the myocardium. Specifically, Late Gadolinium Enhancement (LGE) is the clinical gold standard for the assessment of myocardial viability. In LGE, gadolinium-based contrast agents are administered and retained in scarred tissue about 10 minutes after injection. This leads to shortened *T*_1_ times compared to healthy myocardium and induces a contrast with the surrounding healthy tissue. However, the need for exogenous contrast agents limits its repeated use. Furthermore, its use is contraindicated in patients with renal dysfunction due to the risk of nephrogenic systemic fibrosis (NSF) and recently gadolinium retention in the brain has been observed, raising the level of caution.

The emergence of myocardial parameter mapping offers a promising pathway to contrast-free assessment of myocardial viability. However, initial attempts based on *T*_1_ and *T*_2_-mapping did not provide sufficient contrast for the robust assessment of scar and fibrosis in clinical MRI [1]. *T*_1*ρ*_-mapping has recently been proposed as a promising endogenous contrast alternative for imaging myocardial fibrosis [1] - [6]. *T*_1*ρ*_ is the time constant that characterizes the longitudinal magnetization relaxation in the rotating frame of reference. *T*_1*ρ*_ relaxation is induced by the continuous application of an RF pulse, the so-called spin-lock (SL) pulse [7]. As a result, the magnetization is locked along the RF field, mitigating the loss of transverse magnetization and, thus, suppressing the low frequency contribution to the relaxation that would lower the contrast between normal and infarcted myocardium. SL preparations with different durations are used to generate varying *T*_1*ρ*_ contrast in MRI scans. The data acquired with different *T*_1*ρ*_ weightings are then fit to an exponential decay function to obtain voxel-wise estimation of *T*_1*ρ*_ values.

While promising results were obtained at low field strength [6], the translation of *T*_1*ρ*_-mapping to higher field strengths is hindered by the susceptibility of the SL preparation to $B_{0}$ and $B_{1}^{+}$ field inhomogeneities. Adiabatic SL modules, consisting of amplitude and frequency modulated RF pulses, have the potential to overcome these limitations and allow the use of *T*_1*ρ*_ as an endogenous contrast agent in clinical practice at high fields [8], [9]. In this work, we sought to enable robust in vivo *T*_1*ρ*_-mapping at 3T in a single breath-hold using adiabatic SL preparations. Bloch simulations were performed to optimize the parameter choice for adiabatic preparations. The proposed sequence was then tested on agar-based phantoms and on a healthy volunteer to study the performance of adiabatic and conventional SL preparations in presence of system imperfections.

## II. METHODS

### A. Sequence design

**Fig. 1.**
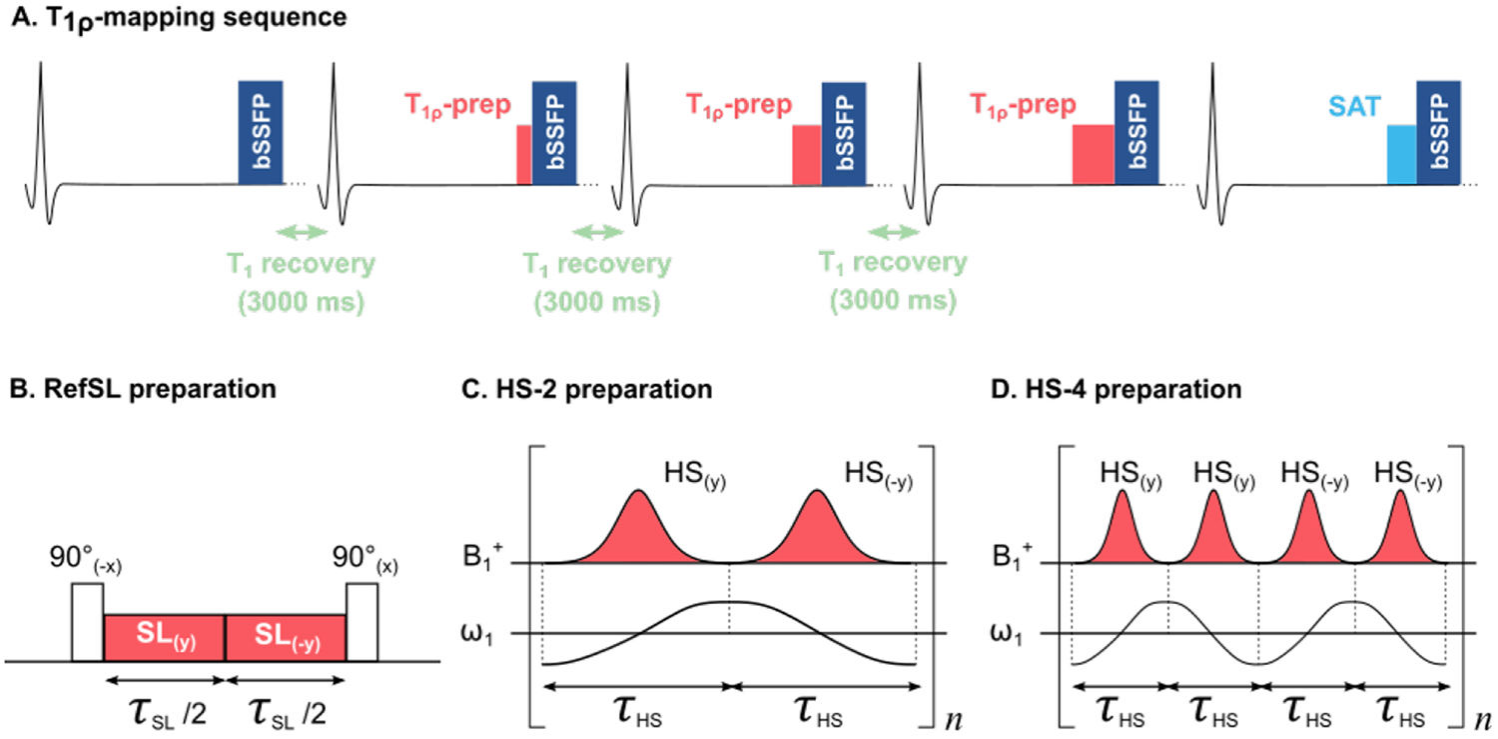
(A) *T*_1*ρ*_-mapping sequence acquiring four *T*_1*ρ*_-prepared images, interleaved with 3 s delay to allow *T*_1_ recovery, and a saturation-prepared image in a single breath-hold. (B) Reference continuous-wave Spin-lock (SL) preparation. (C-D) Adiabatic SL preparations consisting of a train of 2 or 4 HS pulses, with equal total duration.

*T*_1*ρ*_-mapping was performed on a 3T scanner (Ingenia, Philips, Best, The Netherlands). Four *T*_1*ρ*_-prepared images are acquired followed by a saturation-prepared image to approximate infinite SL duration and capture the effects of the imaging readout (Fig. 1). *T*_1*ρ*_-prepared images were acquired with different total SL duration ($\tau_{SL}$ = {0, 60, 120, 180} ms) to induce varying *T*_1*ρ*_ contrast and interleaved with 3 s delay to allow for *T*_1_ recovery. A single-shot balanced Steady-State Free Precession (bSSFP) sequence was used for the acquisition, with the following imaging parameters: Flip Angle = 70°, phantom: resolution = 1 × 1 × 8*mm*^3^, FOV = 250 × 180*mm*^2^, TE/TR = 1.08/2.73 ms, in vivo: resolution = 1.8 × 1.8 × 8*mm*^3^, FOV = 260 × 190*mm*^2^, TE/TR = 0.95/2.25 ms. Cardiac motion compensation was achieved by ECG triggering the acquisition to the mid-diastolic phase. The total scan time was 13 ms, performed during a single breath-hold to mitigate respiratory motion.

### B. *T*_1*ρ*_ preparations

Two adiabatic *T*_1*ρ*_ preparations were compared to a non-adiabatic SL module (RefSL) [6]. The conventional SL module consists of a 90° tip-down pulse followed by an RF pulse with constant amplitude and frequency (duration = $\tau_{SL}$). Finally, a 90° tip-up pulse is applied to restore the longitudinal magnetization. The RefSL block presents additional phase alternation to partially compensate for $B_{0}$ inhomogeneities. Adiabatic *T*_1*ρ*_ preparations, on the other hand, are obtained by concatenating 2 (HS-2) or 4 (HS-4) phase-cycled hyperbolic-secant adiabatic full-passage pulses (Fig. 1b-d) [10]. These pulses are characterized by variable amplitude and frequency, where:

(1) $$B_{1}(t)=B_{1}^{max}\cdot \text{sech}\left(\beta \left(\frac{2t}{\tau_{HS}}-1\right)\right),$$

(2) $$\Delta \omega_{1}(t)=f_{max}\cdot \tanh\left(\beta \left(\frac{2t}{\tau_{HS}}-1\right)\right).$$

The duration of each pulse was determined by the total SL block duration of 60 ms ($\tau_{HS}=30$ ms for HS-2, $\tau_{HS}=15$ ms for HS-4). The peak RF amplitude was set to the maximum ($B_{1,max}^{+}=13.5\mu T$). Bloch simulations of the magnetization evolution for different configurations of adiabatic SL preparations were performed to optimize the remaining parameters β and $f_{max}$. Assuming that an ideal adiabatic preparation yields a final magnetization in alignment with the z-axis, the preparation efficiency was computed through the ratio *M_z_*/*M_z_*(0) of the final and initial longitudinal magnetization, where *M_z_*(0) = 1. Average *M_z_* was computed for every combination of β = 1, 1.25, 1.5, …, 10 and $f_{max}$ = 0, 50, 100, …, 5000 Hz over a design window of ±150 Hz off-resonances and ±25% $B_{1}^{+}$ variations [11].

### C. Phantom and in vivo experiments

The sequence was first tested in a *NiCl*_2_-doped agar-filled vials phantom, submerged in a water bath. Each vial contained a different agar concentration to achieve a range of *T*_1*ρ*_ values. Additionally, *T*_1*ρ*_ dispersion was investigated in phantoms by varying the RefSL amplitude f = 0, 100, …, 500 Hz and HS-2 frequency sweep $f_{max}$ = 250, 350, 500 Hz. Conventional *T*_1_ and *T*_2_ maps were also acquired for phantoms. All measurements were performed with five repetitions in phantoms.

In vivo imaging was performed in one healthy volunteer (female, 21 y.o.). The experiments were approved by the local ethics committee and signed informed consent was obtained prior to scanning.

Both phantom and in vivo images were acquired under two different shimming conditions to test the resilience of the different preparations to system imperfections. Voxel-wise *T*_1*ρ*_ maps were generated for phantom and in vivo scans, using a three-parameter exponential decay model. In vivo *T*_1*ρ*_ maps were segmented to extract the left ventricular myocardium and blood pool.

## III. RESULTS

**Fig. 2.**
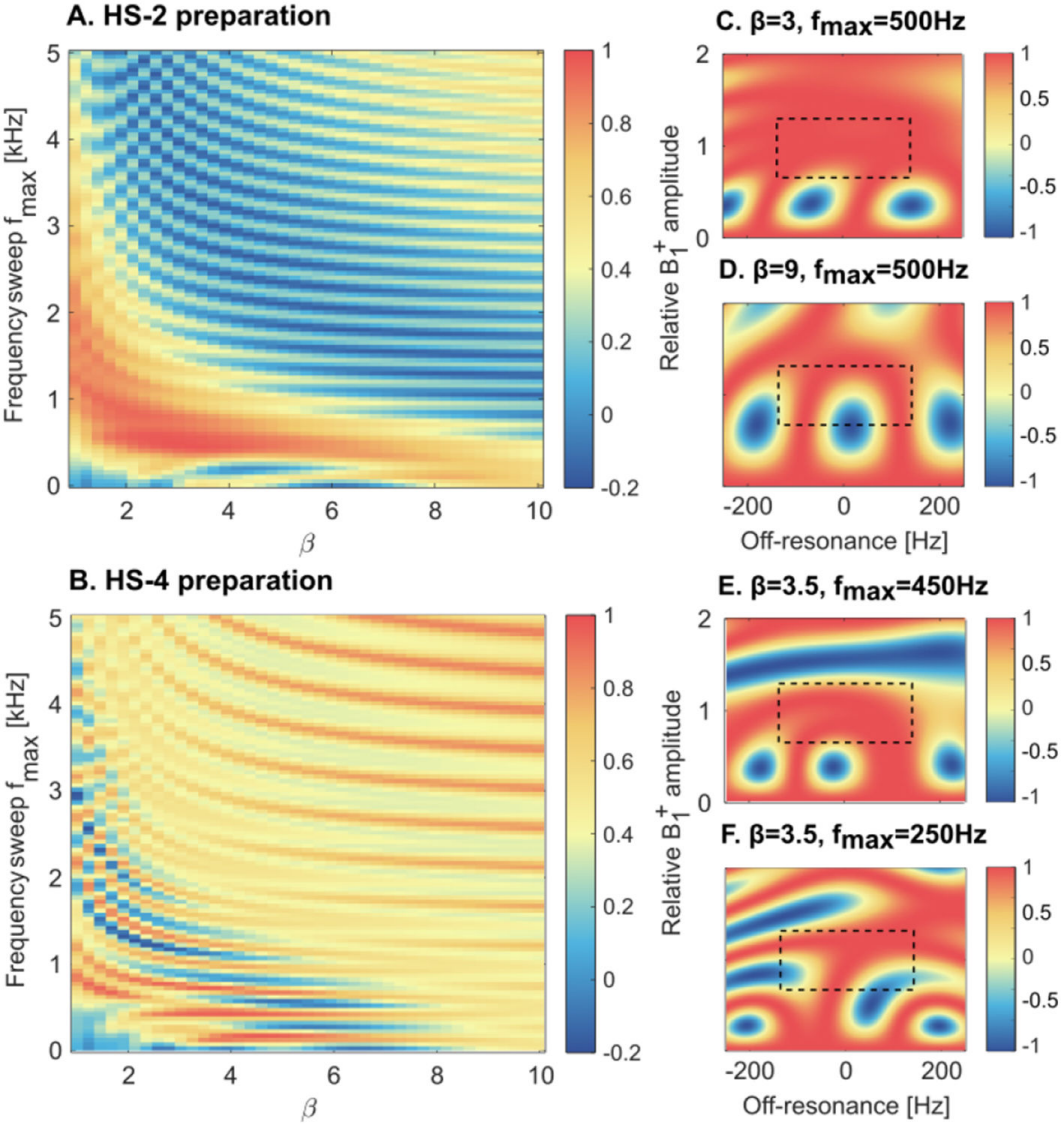
(A-B) HS-2 and HS-4 preparation efficiency, computed for each combination of β and $f_{max}$ values over a design window of ±150 Hz off-resonances and ±25% $B_{1}^{+}$ variations through Bloch simulations. (C-F) Plots showing $B_{0}∕B_{1}^{+}$ imperfections effect on preparation efficiency for exemplary combinations of β and $f_{max}$. Dashed black boxes indicate the design window used to compute average *M_z_* in A-B.

The simulation results for HS-2 and HS-4 preparations averaged over the design window present periodic patterns across β and $f_{max}$ values. For both pulses the best preparation efficiency was obtained for low to intermediate frequency sweep amplitudes and showed an inversely proportional relationship with the parameter β (Fig. 2a-b). Overall, HS-2 shows a higher preparation efficiency in the optimal region than HS-4. Optimal values of {$\beta ,f_{max}$} were chosen as {3,500 Hz} for HS-2 and {3.5, 450 Hz} for HS-4, resulting in average residual magnetization *M_z_* of 0.98 and 0.96, respectively. Examples of preparation efficiency over a range of ±200 Hz $B_{0}$ and 0% – 200% $B_{1}^{+}$ inhomogeneities are shown for selected parameter combinations (Fig. 2c-f).

**Fig. 3.**
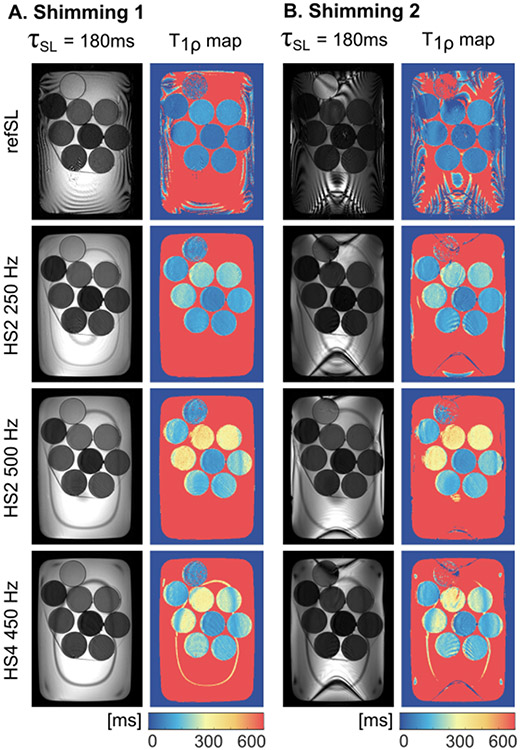
Phantom baseline magnitude images with the longest spin-lock (SL) preparation and corresponding *T*_1*ρ*_-maps. Images are compared for optimal shim region placement (A) and deliberately misplaced shim region (B).

**Fig. 4.**
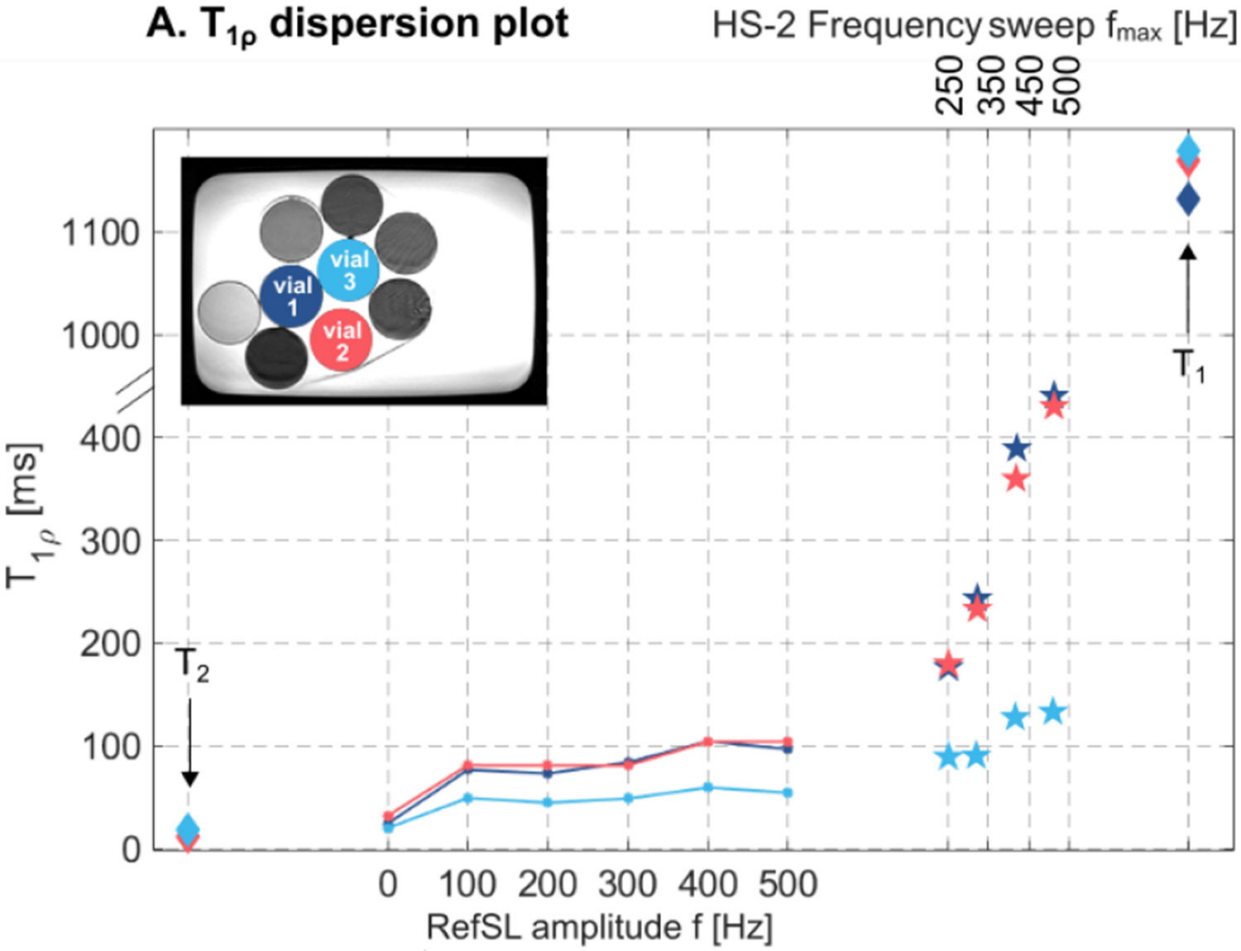
(A) *T*_1*ρ*_ values obtained by varying conventional RefSL amplitude (circle marker) and adiabatic HS-2 frequency sweep amplitude (star marker) compared with *T*_1_ and *T*_2_ values for three different vials.

Fig. 3a shows phantom magnitude images for the longest SL duration and the final *T*_1*ρ*_-maps for RefSL (f = 500 Hz), HS-2 ($f_{max}$ = 250, 500 Hz), and HS-4 ($f_{max}$ = 450 Hz). HS-2 (500 Hz) preparations yield the best results, with fewer artifacts than both HS-4 and RefSL, higher *T*_1*ρ*_ contrast than non-adiabatic preparations, and less susceptibility to the $B_{0}$ shim. The mean ± standard deviation *T*_1*ρ*_ values over the three artifacts-free vials shown in Fig. 4 are vial 1, vial 2, vial 3 = 384.95±40.04, 373.42±39.24, 108.19±9.87 ms for HS-2, 299.31±56.30, 293.12±91.21, 69.66±8.37 ms for HS-4 and 104.96±87.25, 98.12±81.40, 54.64±48.71 ms for RefSL. Non-adiabatic and adiabatic *T*_1*ρ*_ dispersion results in Fig. 4a show consistent trends, with *T*_1*ρ*_ values increasing with f and $f_{max}$, respectively. Overall, adiabatically prepared sequences yield longer *T*_1*ρ*_ times compared with non-adiabatic pulses.

**Fig. 5.**
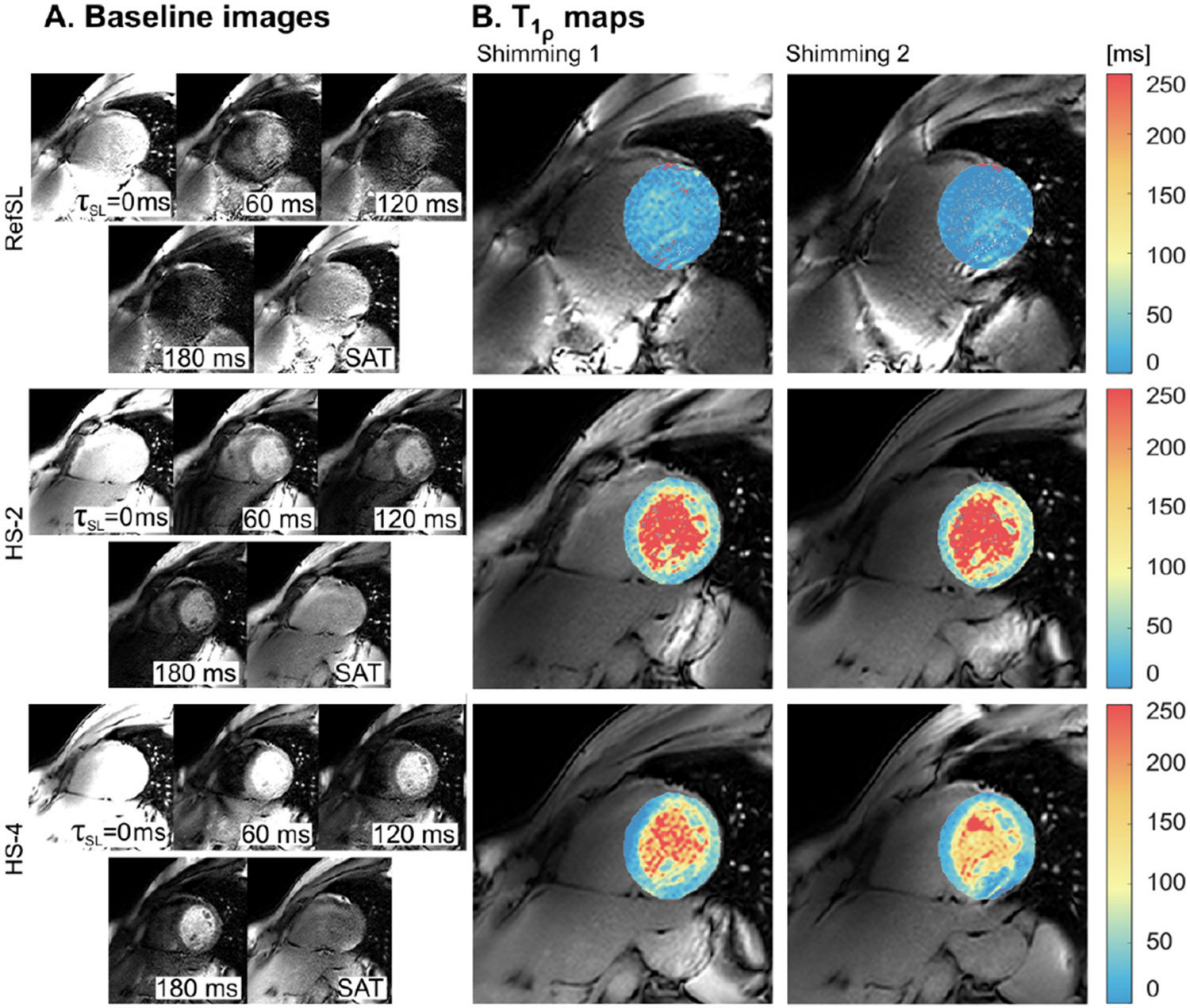
In vivo short-axis baseline magnitude images and corresponding *T*_1*ρ*_-maps obtained from voxel-wise three-parameter exponential decay model fitting for RefSL, HS-2 and HS-4 preparations. *T*_1*ρ*_-maps are segmented to extract left-ventricle myocardium and blood pool and overlaid on the $\tau_{SL}=0$ ms baseline image. *T*_1*ρ*_-maps are compared for two different shimming conditions.

Fig. 5 shows the short-axis *T*_1*ρ*_-weighted baseline images and the corresponding overlaid *T*_1*ρ*_-maps for the myocardium and left-ventricular blood pool of a healthy volunteer. In vivo average *T*_1*ρ*_ values (± standard deviation) in the myocardium were: 16.01 ± 20.75, 148.13 ± 54.08, and 54.72 ± 41.04 ms for RefSL, HS-2, and HS-4 preparations, respectively (Fig. 5). Adiabatically-prepared sequences show significantly lower noise and higher contrast between the myocardium and the blood pool than RefSL. Moreover, HS-4 preparations show more *T*_1*ρ*_ inhomogeneities across the myocardium and significant artifacts for shimming 2. Specifically, the differences in measured myocardial *T*_1*ρ*_ values between the two shimming conditions are 89.74%, 26.91%, 58.03% for RefSL, HS-2, and HS-4, respectively (expressed in % over the average *T*_1*ρ*_ obtained through shimming 1).

## IV. DISCUSSION

In this work, we investigate the use of adiabatic preparations for $B_{0}$ and $B_{1}^{+}$ resilient myocardial *T*_1*ρ*_-mapping at 3T. Our results show improved map quality for adiabatic preparations both in phantom and in vivo, compared to the reference SL implementation. Specifically, adiabatic sequences demonstrate good blood/myocardium contrast and retain image quality across different shim conditions. Compared with the measured effect size of 5.93 times the myocardial *T*_1*ρ*_ std observed at 1.5T [1], our adiabatic method show potential for differentiating between healthy and infarcted myocardium at 3T.

The effective field strength and orientation vary during adiabatic SL preparations. Thus, the relaxation rate changes throughout the preparation module, rather than sampling a uniform *T*_1*ρ*_. Consequently, each adiabatic *T*_1*ρ*_ preparation probes a wider spectrum of frequencies through the adiabatic sweep, compared to mono-frequency conventional SL. This may lead to a different sensitivity profile in pathological remodelling and its clinical value remains to be evaluated.

Simulations were performed to optimize the adiabatic pulse parameters for two or four HS pulses with equal total SL duration. The total SL duration is constrained in practice by the need to efficiently sample the expected *T*_1*ρ*_ ranges in the human myocardium. While two pulses were phase cycled, four pulses allowed for a full Malcolm Levitt (MLEV) scheme [12], [10]. However, Bloch simulations demonstrate that longer 30 ms pulses in HS-2 show larger and more uniform adiabatic regime regions (red areas in Fig. 2) compared with the HS-4 pulses (15 ms each). Furthermore, the adiabatic regime for HS-2 modules is more clearly separated from the $\text{high}-\beta ∕\text{high}-f_{max}$ area. Those areas show artificially high preparation efficiency values, which result from very fast adiabatic sweeps that do not induce any rotation in the magnetization. Accordingly, in phantom experiments, HS-2 preparations show reduced artifacts in the water bath compared to HS-4 preparations. In vivo HS-2 maps are artifact-free for both shimming conditions. HS-4 maps, on the other hand, present residual off-resonance artifacts as well as more inhomogeneous *T*_1*ρ*_ values across the myocardium. These results indicate that longer HS pulses, with slower frequency sweeps, achieve better performance and thus HS-2 preparations are to be preferred over MLEV-cycled HS-4 modules.

## V. CONCLUSIONS

Our results suggest that adiabatic *T*_1*ρ*_-preparations allow for robust in vivo quantification of myocardial spin-lock (SL) relaxation times at high field strengths. This paves the way for potential contrast-free imaging of myocardial fibrosis at 3T.
